# Advancements in Diagnosing Talaromycosis: Exploring Novel Strategies and Emerging Technologies

**DOI:** 10.3390/jof11060434

**Published:** 2025-06-06

**Authors:** Yihui Wang, Xiaoyue Wang, Liyan Xi, Sha Lu

**Affiliations:** Department of Dermatology and Venereology, Sun Yat-Sen Memorial Hospital, Sun Yat-Sen University, Guangzhou 510120, China; wangyh388@mail2.sysu.edu.cn (Y.W.); wxy_1048686617@163.com (X.W.); xiliyan@mail.sysu.edu.cn (L.X.)

**Keywords:** talaromycosis, *Talaromyces marneffei*, diagnosis, monoclonal antibody, molecular diagnostics

## Abstract

Talaromycosis (TM) is an invasive fungal infection caused by *Talaromyces marneffei* (*T. marneffei*). It has high morbidity and mortality rates, particularly among immunocompromised people. Globally, approximately 17,300 cases and 4900 deaths are reported annually. TM often has vague clinical signs with limited current tests, leading to misdiagnosis, incorrect treatments, or the long-term use of expensive antifungal drugs, which raises healthcare costs and patient risks. Although accurate diagnosis is key for starting the right antifungal therapy and improving outcomes, there are not enough reliable and fast tests. Recent progress with monoclonal antibodies (mAbs) that have high specificity for antigens may boost diagnostic accuracy and cut misdiagnosis rates. This review explores current ways to diagnose TM, including culture, histopathology, and molecular methods such as polymerase chain reaction (PCR) and antigen detection. We also discuss the merits and weaknesses of each method and highlight how mAbs may help diagnose TM. We searched PubMed, Web of Science, and Google Scholar for English-language papers (1990—1 January 2025) using “Talaromycosis” OR “*Talaromyces marneffei*” plus diagnostic terms (‘diagnosis’, ‘molecular diagnostics’, ‘monoclonal antibody’, ‘lateral flow’, ‘antigen detection’, and ‘fungal diagnosis’). After deduplication and relevance screening, studies with original data or substantive discussion on *T. marneffei* diagnostics or mAb development were retained to inform this narrative review.

## 1. Introduction

**Figure 1:**
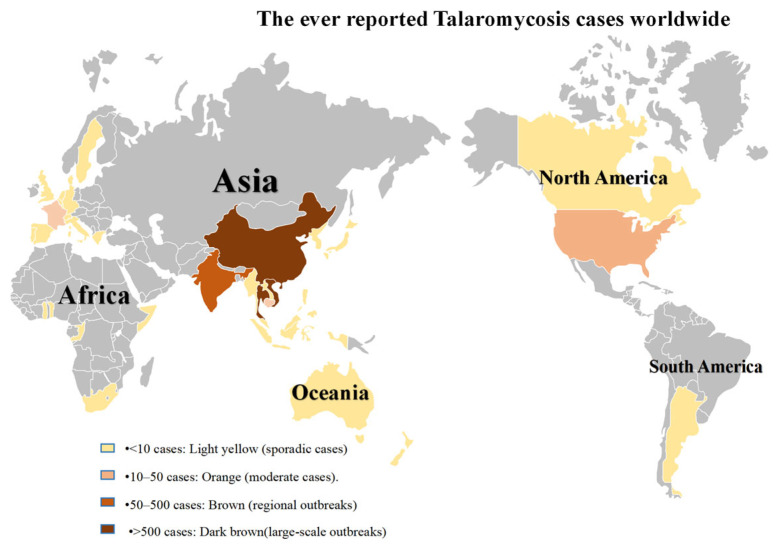
Global distribution of reported TM cases from 1990 to 2024.

Talaromycosis (TM), an invasive fungal disease caused by *Talaromyces marneffei* (*T. marneffei*), has emerged as the third most prevalent opportunistic infection in endemic regions after tuberculosis and cryptococcosis [1,2]. While historically concentrated in Southeast Asia and southern China, shifting environmental patterns and population mobility are driving its geographical expansion, positioning it as a potential global health threat (Figure 1). Diagnostic limitations persist due to reliance on time-consuming culture methods, while therapeutic options remain constrained to toxic antifungal regimens (amphotericin B and triazoles) [3,4]. These factors contribute to a mortality rate of 33.3% [5]. This critical gap in pathogenesis research and diagnostic innovation prompted international researchers in 2021 to advocate for TM’s designation as a “Neglected Tropical Disease” [3], a call reinforced by its recent classification as a medium-priority pathogen on the WHO fungal priority list (WHO fungal priority pathogens list to guide research, development, and public health action).

**Figure 2:**
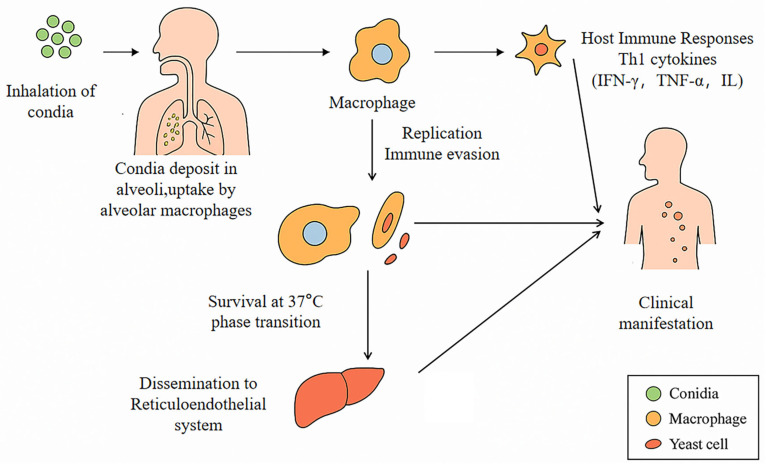
The transmission and pathogenesis of *T. marneffei* infection in humans. (1) Inhalation of spores: Humans frequently inhale conidia from the natural environment via airborne transmission. (2) Alveolar colonization and phagocytosis: Spores are deposited in the alveoli and subsequently engulfed by alveolar macrophages. (3) Phase transition for intracellular survival at 37 °C: Intracellular transformation occurs from filamentous to yeast form, accompanied by temperature adaptation and resistance to lysosomal degradation. (4) Intracellular replication and immune evasion: For instance, through the TUT1-NCOR2/SMRT splicing axis, which inhibits the macrophage inflammatory response and enhances intracellular survival. (5) Dissemination via the reticuloendothelial system: Yeast cells spread to organs such as the liver, spleen, bone marrow, and skin via the bloodstream or lymphatic system. (6) Host response and clinical manifestations: Th1 cytokines (IFN-γ, TNF-α, IL-12) play a critical role in controlling infection. In immunocompromised patients, the infection may progress to diffuse lesions, presenting symptoms such as persistent high fever, anemia, target or umbilical papules on the skin, hepatosplenomegaly, etc.

*T. marneffei’s* infection typically initiates through the inhalation of airborne conidia, which undergo phagocytosis by pulmonary macrophages triggering cell-mediated immune responses (Figure 2) [6]. Emerging data highlight expanding risk populations, including those with primary immunodeficiencies (e.g., IFN-γ autoantibody syndromes), hematologic malignancies, and recipients of prolonged immunosuppressive therapies or organ transplants [7,8].

TM manifests through multiorgan involvement—ranging from respiratory tract lesions to bone, gastrointestinal, and neurological complications—with characteristic painless cutaneous nodules on exposed anatomical regions (face, neck, and limbs) serving as a hallmark presentation [4,6,9,10,11,12]. Primarily, *T. marneffei* infection targeting the lungs, including the bronchi and pleura, presents with predominantly nonspecific and atypical early symptoms [9,10,13]. So, diagnostic imaging, such as chest X-rays and Computed Tomography (CT) scans, suffers from low specificity, often mimicking tuberculosis or other fungal infections, and fails to detect subtle early lesions, resulting in delayed diagnosis. Moreover, imaging changes typically lag behind clinical symptoms and pathological progression, further complicating diagnosis [14]. As the infection advances, *T. marneffei* disseminates via the lymphatic or bloodstream, causing abscess-like lesions in multiple organs. Notably, patients that do not have Human Immunodeficiency Virus (HIV), exhibit distinct clinical patterns: they are less likely to develop skin lesions (44% vs. 71%) or yield positive blood cultures (47% vs. 77%) compared to patients with HIV [15,16]. Consequently, non-HIV patients face significantly longer diagnostic delays (180 days vs. 45 days) and higher mortality rates (29% vs. 21%) [17]. These challenges highlight the limitations of current diagnostic methods, particularly in non-HIV populations, underscoring the urgent need for more sensitive early diagnostic tools and a clearer understanding of optimal diagnostic timing to improve outcomes. This article will provide a comprehensive overview of the diagnostic methods used for *T. marneffei* infection, and discuss their respective goals, advantages and limitations. And in the following review, we have looked at the current ways to diagnose TM, like culture, histopathology, and molecular methods such as polymerase chain reaction (PCR) and antigen detection. Although the focus of this review is on diagnostic innovations, brief reference is made to how faster, more sensitive detection can expedite evidence-based antifungal therapy and thereby improve patient outcomes.

## 2. Literature Search and Study-Selection Strategy

A targeted literature search of PubMed (MEDLINE), Web of Science Core Collection, and Google Scholar was performed for English-language publications dated 1 January 1990 to 1 January 2025. The search strategy combined controlled vocabulary and free-text terms—‘Talaromycosis’ OR ‘*Talaromyces marneffei*’—with diagnostic qualifiers (‘diagnosis’, ‘molecular diagnostics’, ‘monoclonal antibody’, ‘lateral flow’, ‘antigen detection’, and ‘fungal diagnosis’). After duplicate removal, titles and abstracts were screened for relevance, and full texts were reviewed when necessary; studies were included if they reported original data or substantive commentary on diagnostic methods or monoclonal-antibody development for *T. marneffei*.

All records retrieved from PubMed (MEDLINE), Web of Science Core Collection, Embase, Scopus, and Google Scholar were exported in RIS format and imported into Zotero v6.0.30; the program’s built-in duplicate-detection algorithm was applied, and each flagged pair was manually verified before removal. Two authors (Y.W. and X.W.) then independently screened titles and abstracts in Zotero using customized tags to designate “include”, “exclude”, or “uncertain”; full texts for the latter two categories were obtained via institutional access or interlibrary loan and read in Zotero’s PDF viewer, with final eligibility decisions recorded in the same library. Only English-language articles published between 1 January 2000 and 1 January 2025 that presented original data or substantive commentary on *T. marneffei* diagnostic methods or monoclonal antibody development were retained. Papers focused exclusively on therapy, single-case reports, or non-English texts were excluded.

The resulting evidence base underpins the narrative synthesis presented herein.

## 3. Current Diagnostic Methods: Strengths and Limitations

### 3.1. Histopathologic Visualization and Culture (The Gold Standard)

Firstly, various microscopic detection methods are available, including laboratory staining of tissue structures or the examination of cytological specimens. Histopathological sections stained with Grocott-methenamine silver, or periodic acid-Schiff reveal characteristic round to oval *T. marneffei* yeast cells, which exhibit division through cross-wall formation within macrophages [18]. For specimens obtained via fine-needle aspiration, such as lymph node samples, bone marrow aspirates, skin touch preparations, or lymph node biopsies, Wright staining demonstrates distinctively basophilic, spherical, oval, and elliptical yeast cells with prominent central septa, allowing for the diagnosis of *T. marneffei.* However, 30–60% of patients with TM exhibit no cutaneous manifestations [15,19], and the positivity rate of fungal microscopy is relatively low, influenced by multiple factors, including sampling methods and the proficiency of laboratory personnel in slide interpretation.

Culture remains the gold standard for diagnosing *T. marneffei* in clinical practice. This pathogen’s unique dimorphic growth pattern provides distinctive visual characteristics that aid in its identification. When cultured at 25 °C on Sabouraud glucose agar, it produces a greenish-yellow mycelium and secretes a characteristic diffusible red pigment. At 37 °C on brain–heart infusion agar, it transforms from mold to yeast form, a hallmark of its thermally dimorphic nature [20]. According to the data from different specimens, such as sputum (34%), blood (76%), skin biopsy (90%), bone marrow (100%), and lymph node biopsy (100%), the detection accuracy for laboratory culture can be as high as 100% [12]. Despite this high accuracy, this method has limitations. Prolonged incubation (7–14 days) can delay diagnosis and treatment [21]. Additionally, sensitivity varies among patient populations and sample types; for instance, blood cultures may fail to detect the pathogen in up to 50% of cases, often identifying TM only at advanced stages when the infection has disseminated extensively [15]. These challenges highlight the critical need for more rapid and sensitive diagnostic methods. Enhanced diagnostic tools, such as molecular testing or antigen detection, could complement traditional culture techniques, addressing their limitations and supporting better patient care.

### 3.2. Serology Test

A variety of antigen or antibody detection methods have been used for the diagnosis of *T. marneffei* infection. Firstly, the enzyme-linked immunosorbent assay (ELISA) was designed based on the monoclonal antibody (mAb) EB-A1 prepared from the galactomannan (GM) antigen of the fungal cell wall [22,23]. Studies show that the GM optical density (OD) index increases in HIV-negative talaromycosis patients, though the median OD value is lower than in HIV-positive patients. GM testing can aid in diagnosing talaromycosis in HIV-negative patients and improve early diagnostic accuracy when combined with real-time quantitative PCR [24], and GM determination cross-reacts with other fungal species like *Aspergillus* and *Cryptococcus*, limiting its specificity. Antigens derived from *T. marneffei*, including immunogenic yeast proteins (200, 88, 54, and 50 kDa) [25], a 38 kDa mycelial protein [26], and cytoplasmic yeast proteins (61, 54, and 50 kDa) [27], can elicit human immune responses. These antigens are potential candidates for the development of specific antibodies to diagnose disseminated *T. marneffei* infections. Sirida et al. developed an immunochromatographic strip test (ICT) using a liquid-phase sandwich immunoassay to detect *T. marneffei* in urine. The method employs *T. marneffei*-specific mAb4D1 conjugated with colloidal gold nanoparticles as the signal reporter. Galanthus nivalis agglutinin (GNA) is used as the test line on the nitrocellulose membrane [28]. The ICT based on mAb4D1–GNA showed specific binding to the yeast phase antigen of *T. marneffei*, with no reaction to other common pathogenic fungi. It was validated using 341 urine samples, achieving 89.47% sensitivity, 100% specificity, and 97.65% accuracy. However, due to low yeast phase antigen levels in urine, the sensitivity in patients with negative blood cultures may be reduced. Additionally, the ICT is not suitable for serum samples, as the serum matrix interferes with the colloidal gold conjugate, leading to colloidal gold degradation and liquid blockage, reducing diagnostic sensitivity to 71.43% (15/21).

Yuan et al. identified that the Mp1p protein, encoded by the *MP1* gene, is a prevalent cellular mannoprotein found in the cell walls of yeast, hyphae, and conidia of *T. marneffei* [29]. This protein exhibits a strong affinity for concanavalin A. The Mp1p antigen is secreted in substantial quantities and serves as a critical virulence factor for *T. marneffei* [29]. Currently, anti-Mp1p mAb is primarily used for antigen detection enzyme immunoassays (EIAs). Chen et al. conducted a prospective cross-sectional study using culture and/or pathology-confirmed penicilliosis as the gold standard to evaluate the diagnostic accuracy of EIAs, GM assays, and blood cultures performed within 3 days of hospitalization [30]. In this study, the positive rate of EIA detection in penicilliosis patients was similar to blood culture (72% vs. 81.7%, *p* = 0.122) and consistent with the gold standard (kappa: 0.729). The sensitivity, specificity, PPV, and NPV of Mp1p antigen detection were 72.0%, 96.8%, 91.8%, and 87.6%, respectively, outperforming GM detection. Wang et al. detected Mp1p antigen and antibody in serum from 20 TM patients and 525 negative controls [31,32]. The Mp1p antigen ELISA showed 75% sensitivity, outperforming the 30% sensitivity of the Mp1p antibody test. A Vietnamese study also reported 86.3% sensitivity and 98.1% specificity for Mp1p antigen detection in diagnosing TM in patients with HIV [33]. Moreover, multiple studies by Zheng J [34], Prakit K [35], Ning C [36], and others, have confirmed the effectiveness of various serological diagnostic methods for Mp1p. In light of these promising results, the mAb-Mp1p enzyme immunoassay is currently being evaluated in one multicenter prospective study (NCT04033120) as a rapid diagnostic test for *T. marneffei* diagnosis [33].

### 3.3. Molecular Detection

Contemporary molecular diagnostics for *T. marneffei* detection leverage diverse amplification techniques, including real-time quantitative PCR, nested PCR, semi-nested PCR, and rolling circle amplification [37], targeting species-specific genomic loci such as 5.8S rRNA, 18S rRNA, and *MP1* gene regions [38,39,40]. These methods demonstrate variable sensitivity (10–70%) but robust specificity (95%) for rapid pathogen identification [1]. Notably, advances in molecular assays have expanded their clinical utility for fungal diagnostics, including *T. marneffei* detection. The real-time fluorescence quantitative PCR designed by Sha Lu et al. for detecting *T. marneffei* DNA demonstrated a sensitivity of 77% [37]. Other rapid PCR-based detection methods include the TaqMan real-time fluorescence quantitative PCR targeting the *MP1* gene, introduced by Hien et al., which exhibited a sensitivity of 60% and a specificity of 100%, with a detection time of only 5–6 h [40]. Additionally, Pornprasert et al. utilized the 5.8S ribosomal DNA TaqMan real-time fluorescence quantitative PCR method, achieving a PCR sensitivity of 10 yeast cells/mL in seeded blood and completing the detection within 1 day10 [41].

Metagenomic next-generation sequencing (mNGS) has emerged as a transformative tool, particularly for diagnosing opportunistic infections in immunocompromised hosts. Retrospective data reveal that mNGS achieves 100% sensitivity and 98.7% specificity for *T. marneffei* detection within 26 h, outperforming conventional methods in identifying polymicrobial or atypical presentations [42,43]. Li et al. reported that mNGS had a diagnostic sensitivity of 89% and specificity of 74% compared to culture, with an overall concordance of 78%. Compared to smear and PCR, mNGS showed a sensitivity of 78% and specificity of 70%. In cases where culture results were negative, mNGS diagnosed 64% of immunocompromised HIV-negative patients and 28% of immunocompetent patients with streptotrichosis. This may be due to complex medication histories and the presence of multiple pathogens affecting culture sensitivity [44]. Additionally, mNGS can detect mixed infections involving rare pathogens and guide decisions on discontinuing antimicrobial therapy [45,46]. So, clinical mNGS testing has improved diagnostic rates for invasive fungal diseases (IFD) in the lungs [47], bloodstream [48], and central nervous system [49], as well as identified uncommon or unexpected fungal pathogens [50].

Matrix-assisted laser desorption/ionization time-of-flight mass spectrometry (MALDI-TOF MS) is an innovative technology applied in clinical microbiological examinations. Because of its simplicity, high accuracy, short turnaround time, and cost-effectiveness, MALDI-TOF MS has been used for fungal identification, especially for atypical *T. marneffei* strains [51,52]. Xu et al. reported that MALDI-TOF MS accurately identified all 135 *T. marneffei* isolates, yielding a 100% identification rate [53]. In practice, the automated MALDI-TOF workflow offers high throughput and minimal hands-on time: it can differentiate *T. marneffei* from related fungi and generally achieve species-level identification within minutes after culture [54].

### 3.4. Immunity-Guided Predictive Diagnosis of T. marneffei

Recent insights into how the immune system responds to *T. marneffei* have made it easier to catch infections early and predict how severe they might become. In children who are HIV-negative, certain immune characteristics—like unusual cytokine levels or specific genetic issues—help doctors identify suspicious *T. marneffei* infections. A practical approach involves step-by-step flow cytometry testing to check for primary immunodeficiencies linked to the Interferon-γ (IFN-γ)/*STAT1* pathway, making it simpler to figure out whether the HIV-negative children might be at risk [55]. Patterns in immunoglobulins also provide helpful clues. For example, high levels of IgM but low IgG and IgA, or extremely high levels of IgE, can point to CD40L deficiencies or *STAT3* gene mutations [55]. When *STAT1* genes are overactive, they stay phosphorylated too long after IFN-α or IFN-γ stimulation, helping doctors spot a gain-of-function *STAT1* mutation. An IFN-γ receptor deficiency, on the other hand, shows up as an abnormal *STAT1* response to IFN-γ but normal reactions to IFN-α. Therefore, Wang et al. have suggested that the level of serum IFN-γ may be a simple and rapid method to diagnose *T. marneffei* infection in HIV-negative patients [3]. At the same time, machine learning and data from large patient groups have led to prediction models that combine common clinical and lab results to gauge infection risk and outcomes. Studies have shown that skin lesions, AST levels, the ALT/AST ratio index (AARI), peripheral or abdominal lymphadenopathy (POAL), and CD4+ T-cell counts can serve as strong indicators. Notably, a random forest model using mainly AST and AARI data has shown impressive accuracy, proving that even straightforward measures can be extremely useful for early diagnosis [56]. For example, Qin et al. [57] have found that age, AST/ALT ratio, albumin levels, and blood urea nitrogen levels can predict the risk of death in patients with HIV with TM. New approaches that add more immune markers, advanced immunophenotyping, and comprehensive multi-omics analysis aim to make *T. marneffei* detection even more precise and faster.

Ongoing studies continue to refine these strategies. Incorporating additional immune biomarkers, advanced immunophenotyping, and multi-omics data is expected to enhance the precision and timeliness of *T. marneffei* infection identification. Host-based immunological insights, genetic profiling of immunodeficiencies, and innovative machine learning tools are establishing a foundation for rapid, reliable, and personalized diagnosis of *T. marneffei* infection in HIV-negative patients.

### 3.5. Emerging Strategies and Technologies

Building on the strengths and limitations of the four diagnostic modalities discussed above, along with recent advances in mAb-based assays, it seems that mAb have unlimited potential in the diagnosis of *T. marneffei*. Studies have shown that the immunohistochemistry (IHC) method based on monoclonal antibodies can effectively distinguish *Aspergillus* from other filamentous fungi, which is helpful for the clinical diagnosis and treatment of Invasive Aspergillosis (IA) [58]. For example, formalin-fixed and paraffin-embedded lungs, liver and skin tissues, and mAb EB-A2 can accurately identify *Aspergillus* hyphae and fungal fragments within phagocytes [59,60]. In addition, compared with traditional culture techniques, this technology not only has a faster recognition speed, but also can detect antigens released or metabolized into parenchymal tissues through mAb probes for IHC/IF detection [61].

In another field, multifunctional nanoprobes can combine targeted drug delivery, therapy, dual-modal molecular imaging, and therapeutic monitoring. They enable precise diagnosis and treatment under image guidance, expanding into other disease areas like fungal infections. Davies et al. developed the humanized mAb JF5 (hJF5) from the mouse mAb JF5 (mJF5). hJF5 binds to the *Aspergillus* fumigatus galactofuranose antigen, present only during active pathogen growth, distinguishing inactive spores from invasive hyphae in infected lungs. Conjugated with [64Cu]-NODAGA, the [64Cu]-NODAGA-hJF5 tracer is ideal for sequential imaging of invasive pulmonary aspergillosis due to its long half-life, making it suitable for repeated imaging after a single injection. Studies also showed that the antigenic determinant β1,5-galactofuranose (Galf) bound by mJF5 and hJF5 is unique to the target mannoprotein antigen and absent in mammalian carbohydrates. It ensures high specificity in imaging and reduces adverse reactions combining with hJF5’s high specificity [62]. These precedents underpin our proposal that analogous mAb-driven platforms could markedly shorten the diagnostic window for talaromycosis.

The most advanced *T. marneffei*-specific mAbs provide a ready source of high-affinity reagents that can be repurposed for diagnostic assay development. The first line for defending against microbial infection is achieved by activating pro-inflammatory mediators to signal and attract immune components to the infection site [63]. Some of the pro-inflammatory mediators are lipid molecules derived from arachidonic acid (AA). As a key mediator, AA regulates the production of inflammatory factors like Monocyte Chemotactic Protein 1 (MCP-1), Tumor Necrosis Factor (TNF), and Interleukin (IL), playing a crucial role in both homeostasis and inflammation. Kwok-Yung Yuen’s team found that Mp1p-LBD1/2 has a monomer structure of a five-helix bundle with a long hydrophobic central cavity, which binds intracellular fatty acids like AA with high affinity. Quantitative LC-MS analysis showed that J774 cells infected with wild-type *T. marneffei* had lower AA concentrations and reduced production of downstream AA metabolites, IL-6, and TNF-α compared to those infected with *MP1* knockout *T. marneffei* [64]. Additionally, the ability of Mp1p-LBD1/2 to bind one or two AA molecules with varying affinities reflects its flexibility in responding to host-produced inflammatory mediators. Based on the above findings, future studies can actively explore *T. marneffei*-specific antibodies and integrate them with advanced technologies such as nanomaterials and radioactive nuclides. This combined approach will advance the diagnosis of *T. marneffei* infections and has the potential to open new avenues for the treatment of *T. marneffei*. These developments hold significant clinical and social significance by enhancing early diagnosis and enabling more precise treatments for infections caused by this pathogen.

## 4. Discussion

**Figure 3:**
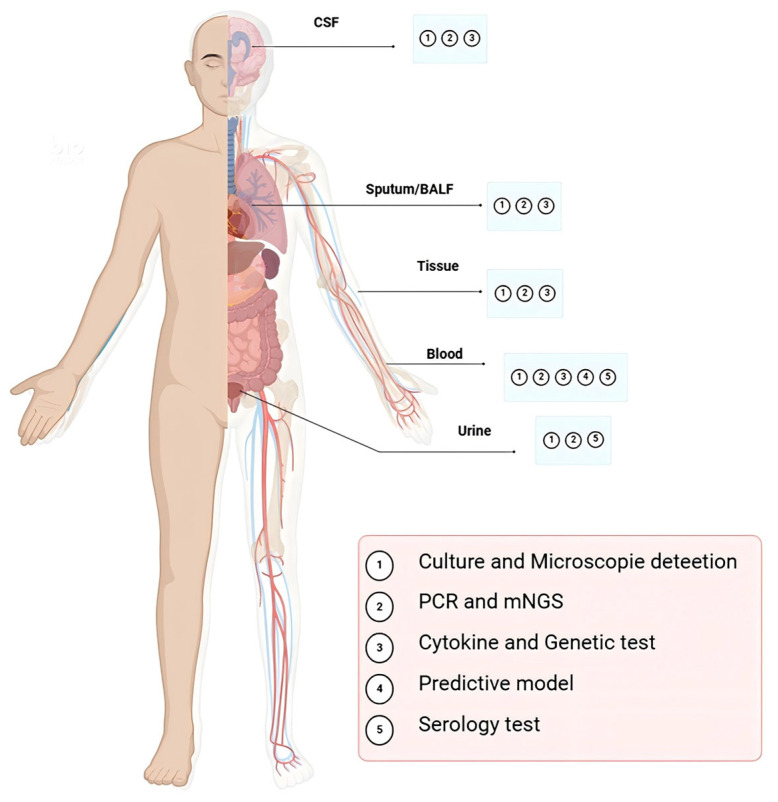
A comprehensive summary of existing diagnostic methods for TM based on various sample sources.

This review focuses on current diagnostic strategies for TM and summarizes existing methods based on various sample sources (Figure 3). Diagnostic approaches range from traditional culture and microscopy to advanced molecular techniques, each with distinct strengths and limitations (Table 1).

Culture remains the gold standard due to its nearly 100% specificity (isolating the live organism), but it is time-consuming and can be insensitive in early or localized disease. Blood or bone marrow cultures take ~1–2 weeks to grow and yield positive results in only ~60–75% of disseminated cases (meaning up to ~40% of infections may be missed). Histopathology offers a quicker presumptive diagnosis by visualizing the characteristic fission yeast cells of *T. marneffei* in clinical specimens. Its specificity is high when classic features (oval yeasts with a transverse septum) are recognized, but sensitivity is moderate and heavily dependent on tissue burden and sample site. Notably, 30–60% of patients lack the hallmark skin lesions, limiting opportunities for rapid smear diagnosis. Histopathology also requires invasive biopsies and skilled personnel, which may not be readily available in all settings.

Antigen detection assays have substantially improved early diagnosis. The galactomannan (GM) ELISA, originally developed for aspergillosis, often cross-reacts with *T. marneffei*. It shows moderate sensitivity (~80%) but only around 80% specificity, as Talaromyces GM antigen can produce false positives in the presence of other fungi. In contrast, an ELISA targeting the *T. marneffei*-specific Mp1p mannoprotein antigen offers higher accuracy. The Mp1p antigen ELISA has reported sensitivities in the 72–85% range and specificity ~95–99%, markedly outperforming blood culture in early disease. However, their availability outside endemic areas is limited, and GM tests may be used as a surrogate in those settings despite the risk of cross-reactivity.

Molecular techniques provide another leap in diagnostic performance. Polymerase chain reaction (PCR) assays—including conventional, nested, and real-time TaqMan formats—targeting specific fungal DNA (e.g., 5.8S or 18S rRNA, or the *MP1* gene) achieve very high specificity (>95%). Reported sensitivities have ranged widely with methodology, but optimized quantitative PCR can detect ~70–88% of infections, substantially higher than culture. The main drawbacks are cost and infrastructure: PCR testing requires specialized laboratories and may not be available in many resource-limited hospitals. Moreover, false negatives can occur if fungal DNA is low or inhibitors are present. So, a negative PCR does not entirely rule out talaromycosis. Metagenomic next-generation sequencing (mNGS) represents an emerging high-tech solution, offering unbiased pathogen detection. In recent studies, mNGS has shown outstanding performance for *T. marneffei*, with sensitivities approaching 95–100% and specificity ~98–99%. Notably, mNGS can identify cases that are missed by culture or histology; mNGS can be applied to virtually any sample type (blood, tissue, and even paraffin-embedded biopsies). The trade-offs are the high cost and limited accessibility: currently this technology is mainly available in tertiary centers or via send-out services, and its complexity and expense make it impractical as a first-line test in many endemic areas.

MALDI-TOF mass spectrometry has enhanced the clinical utility of culture by enabling rapid species identification. Once a fungus is grown, MALDI-TOF can confirm *T. marneffei* within minutes by its proteomic fingerprint, even by relatively inexperienced staff. This significantly shortens the time to definitive identification compared to morphology-based methods. The limitation is that MALDI-TOF requires a viable isolate and a reference database entry for *T. marneffei*. In practice, the database for this rare pathogen is still being refined. In endemic centers with up-to-date libraries, MALDI-TOF provides near-100% identification accuracy, but in non-endemic labs misidentification it is possible if the system has not been validated for *T. marneffei*. Moreover, MALDI-TOF’s benefit is null until culture grows, so it does not alleviate the initial diagnostic delay.

In summary, each diagnostic modality plays a role in talaromycosis management. Traditional culture and histopathology offer definitive confirmation but are slow and may miss early infections. Antigen assays (Mp1p and GM) and PCR-based tests greatly improve early sensitivity and speed, aiding prompt therapy, though they require specific reagents and laboratory capacity. mNGS provides the most comprehensive and rapid detection—important for atypical or disseminated presentations —but its high cost and limited availability constrain routine use. MALDI-TOF, while not a primary diagnostic, streamlines laboratory confirmation once an isolate is obtained. Clinicians in endemic areas often employ a combination of these methods to balance accuracy and timeliness, and the optimal approach may differ in non-endemic regions where access to specialized tests is limited.

On the other hand, machine learning using clinical and immunological data offers a novel approach to enhance diagnosis accuracy by integrating clinical and immunological data, such as biomarkers, immunoglobulin levels, and clinical indicators, to assess infection risk and predict outcomes. Yet, reliance on similar datasets questions their effectiveness across diverse populations. More multi-center studies are needed to enhance reliability. Monoclonal antibodies targeting *T. marneffei*-specific antigens, such as Mp1p, have significantly improved diagnostic accuracy due to their high specificity and sensitivity in detecting fungal components. Nanomaterials and multifunctional nanoprobes also show great potential in the diagnosis and treatment of talaromycosis. When combined with monoclonal antibodies, they may significantly improve early diagnostic accuracy.

Notably, the incidence of talaromycosis among HIV-negative patients is progressively rising. Furthermore, enhanced population mobility introduces growing challenges to regions and countries that are traditionally non-endemic for this disease. Consequently, in clinical settings, nonspecific symptoms indicative of *T. marneffei* infection should not lead to reduced vigilance simply because a patient is HIV-negative. For patients with immunodeficiency, organ transplantation, malignancies (particularly hematologic malignancies), or other risk factors outlined in this review, advanced diagnostic tools, such as mNGS, TaqMan real-time fluorescence quantitative PCR, MALDI-TOF MS, and serological testing, should be promptly employed when talaromycosis is suspected. These technologies play a pivotal role in facilitating the early diagnosis and effective management of *T. marneffei* infections.

## Figures and Tables

**Table 1:** A comprehensive overview of the diagnostic methods employed for *T. marneffei* infection.

Diagnosis Approach	Target	Advantage	Disadvantage	Turnaround Time	Approximate Cost	Reference
Culture and microscopic detection	Blood, Skin, Bone marrow, and CSF ^a^	Gold standard; Pathologic changes in the tissue; Morphology of the *T. marneffei*	Requires pure, uncontaminated fungal isolates for reliable results; Too slow, delays therapeutic intervention, and limited sensitivity(disseminated infection)	7–14 days (up to 2–4 weeks)	Low	[16,19,20,21,22]
Serology test	Blood and Urine	Easily performed on readily accessible samples	False-negative in immunocompromised host	Hours to 1 day	Low	[4,23,24,28,29,30,31,32,33,34]
PCR and mNGS	Blood, Skin, Bone marrow, and CSF ^a^	Various sample resources; Platform widely available; Rapid turnaround time	Lack of standardization; Contamination can be problematic	Within 1–2 days	High	[14,17,39,40,41]
Cytokine and Genetic test	IgM, IgG, IgA and IgE; IFN-α/γ; CD40L, *STAT*1/3	Various sample resources; Rapid turnaround time	Lack of standardization; Unknown pathogen species; Unknown infection site	Within 1–2 days	Moderate	[3,57]
Predictive model	Age, AST/ALT ratio, Albumin levels, and BUN ^b^ levels	Predict the risk of death in patients with HIV with talaromycosis	Lack of standardization	Immediate (once labs available)	Low	[59]

^a^ CSF, Cerebral Spinal Fluid, ^b^ BUN, Blood Urea Nitrogen.

## Data Availability

The data presented in this study are available in WHO at https://www.who.int/publications/i/item/9789240060241 (accessed on 15 February 2024).

## Abbreviations

The following abbreviations are used in this manuscript:

TM	Talaromycosis
IA	Invasive Aspergillosis
HIV	Human immunodeficiency virus
mAbs	monoclonal antibodie
PCR	polymerase chain reaction
CT	Computed Tomography
NGS	Next-Generation Sequencing
FFPE	Formalin-Fixed and Paraffin-Embedded
POCT	Point-of-Care-Testing
EIAs	Antigen-Detection Enzyme Immunoassays
IFN	Interferon
STAT1	Signal Transducerand Activator of Transcription 1
AARI	ALT/AST Ratio Index
POAL	Peripheral Or Abdominal Lymphadenopathy
ELISA	enzyme-linked immunosorbent assay
GM	Galactomannan
OD	Optical Density
ICT	Immunochromatographic Strip Test
GNA	Galanthus Nivalis Agglutinin
IHC	Immunohistochemistry
AA	Arachidonic Acid
